# Environmental DNA reveals tropical shark diversity in contrasting levels of anthropogenic impact

**DOI:** 10.1038/s41598-017-17150-2

**Published:** 2017-12-04

**Authors:** Judith Bakker, Owen S. Wangensteen, Demian D. Chapman, Germain Boussarie, Dayne Buddo, Tristan L. Guttridge, Heidi Hertler, David Mouillot, Laurent Vigliola, Stefano Mariani

**Affiliations:** 1 Ecosystems & Environment Research Centre, School of Environment & Life Sciences, University of Salford, Salford, M5 4WT UK; 2 Department of Biological Sciences, Florida International University, 11200 S.W., 8th Street, Miami, Florida 33199 USA; 3 MARBEC, UMR IRD-CNRS-UM-IFREMER 9190, Université Montpellier, Languedoc-Roussillon, 34095 Montpellier Cedex France; 4 IRD (Institut de Recherche pour le Développement), Laboratoire d’Excellence Labex Corail, UMR IRD-UR-CNRS ENTROPIE, Centre IRD de Noumea, BP A5, 98800 Noumea Cedex, New Caledonia France; 5 University of the West Indies, Discovery Bay Marine Laboratory and Field Station, P.O. Box 35, Discovery Bay, St. Ann Jamaica; 6 Bimini Biological Field Station Foundation, South Bimini Bahamas, Bahamas; 7 The SFS Centre for Marine Resource Studies, Turks and Caicos Islands, UK

## Abstract

Sharks are charismatic predators that play a key role in most marine food webs. Their demonstrated vulnerability to exploitation has recently turned them into flagship species in ocean conservation. Yet, the assessment and monitoring of the distribution and abundance of such mobile species in marine environments remain challenging, often invasive and resource-intensive. Here we pilot a novel, rapid and non-invasive environmental DNA (eDNA) metabarcoding approach specifically targeted to infer shark presence, diversity and eDNA read abundance in tropical habitats. We identified at least 21 shark species, from both Caribbean and Pacific Coral Sea water samples, whose geographical patterns of diversity and read abundance coincide with geographical differences in levels of anthropogenic pressure and conservation effort. We demonstrate that eDNA metabarcoding can be effectively employed to study shark diversity. Further developments in this field have the potential to drastically enhance our ability to assess and monitor elusive oceanic predators, and lead to improved conservation strategies.

## Introduction

Oceanic ecosystems are increasingly impacted worldwide. Marine predators are under often unsustainable fishing pressure, which has resulted in several documented cases of stock collapses^1–3^. Elasmobranch (sharks and batoids) populations specifically have suffered from overexploitation and stock declines^4–9^. They are key species in virtually all marine trophic webs^10,11^ and have long been in conflict with human societies, due to their perceived competition with fishers^12^ or hazardous nature^13,14^. Owing to their relatively slow growth rate and low fecundity^15^, they are also particularly vulnerable to overfishing^16–18^. Only recently have elasmobranchs become the focus of conservation initiatives^4,19^, as the importance of these charismatic animals for the maintenance and resilience of healthy ecosystems is widely acknowledged^20–23^.

The development of management strategies for elasmobranchs depends on accurate population assessments in the field. Yet, currently established survey methods, such as fishing by long-lining or gill-netting, acoustic monitoring, baited remote underwater video (BRUV), underwater visual census (UVC) and fisheries-dependent population surveys, are often resource intensive, selective and dependent on taxonomic expertise, and sometimes invasive and potentially traumatogenic^24–26^. Therefore, biologists and managers worldwide are faced with considerable challenges due to the high effort and cost associated with the assessment and monitoring of elasmobranch biodiversity, abundance and distribution.

Environmental DNA (eDNA), DNA isolated directly from environmental samples such as soil or water, can be amplified, sequenced and assigned back to its species of origin through (meta)barcoding and has been suggested as an alternative to track species presence and abundance in their environment^27,28^. Due to its limited persistence in the water column — in seawater even small (100-bp) eDNA fragments degrade beyond detectability within days^29^— the detection of eDNA from a specific taxon indicates its recent presence in the environment^30–32^. Accordingly, over the past couple of years eDNA methods have increasingly been applied for the detection of rare and invasive species^33–35^. Moreover, it has been demonstrated that eDNA metabarcoding has the ability to outperform traditional survey methods for diverse taxa, including teleost fish, both in freshwater^36–39^ and in marine ecosystems^40–42^.

The first reported study to detect elasmobranch eDNA in natural water samples employed DNA barcoding (aiming to detect a single species in the environment), for the detection of the largetooth sawfish (*Pristis pristis*)^25^. Similarly, a species-specific approach was recently applied to amplify whale shark (*Rhincodon typus*) eDNA from oceanic water samples^42^. On the other hand, eDNA metabarcoding has the potential to simultaneously identify several taxa from an environmental sample^28^, which is clearly essential for community-level assessments. However, previous studies have encountered challenges concerning elasmobranch specific detection, when applying this multispecific approach^43,44^. And although three species of elasmobranch have recently been detected in a large-scale marine eDNA study using a primer set designed for teleosts^45^, we are still lacking evidence that eDNA metabarcoding can successfully be applied to describe elasmobranch diversity across a range of natural settings, for the purpose of ecosystem assessment and management.

Here, for the first time, we employ eDNA metabarcoding of natural seawater samples to specifically investigate shark communities in Atlantic and Pacific tropical ecosystems, using a previously published primer set targeting a 127 bp stretch of the mitochondrial COI region^46^. We assess the potential of this low-effort approach for multi-species elasmobranch detection, and specifically examine whether patterns of species diversity and eDNA read abundance, reflect the known degree of anthropogenic impact in two independent tropical marine systems.

In the greater-Caribbean, there has been a long and ongoing history of elasmobranch exploitation, and high anthropogenic pressure in coastal zones has led to the broad-scale depauperation of elasmobranchs on Caribbean reefs^8,47^. However, many species do still occur in populated areas where strong fishing regulations are in place or where specific shark conservation policies have been enacted^8^. In the Bahamas, for instance, gillnet and long-line fishing have been prohibited since 1991 and their national waters have been declared a shark sanctuary in June 2011, prohibiting directed fishing or even the retention of shark by-catch^48^. In the wider Indo-Pacific region, overfishing and poaching are also responsible for declines in elasmobranch populations^49^; nevertheless, elasmobranchs do still occur in relatively high numbers around remote, isolated locations such as coral reefs on uninhabited atolls in the northern Line Islands^50^ and the Chagos Archipelago^51^. Furthermore, although several widely-distributed elasmobranch species are in effect cosmopolitan or circum-tropical, significant biogeographical differences exist between the Caribbean and the Pacific Coral Sea, which allows also for a broad-scale eDNA comparison of community composition.

Elasmobranch species inventories and assessment of geographical distributions based on eDNA metabarcoding could potentially represent an important tool for rapid environmental monitoring and hence influence conservation management and policy decisions. This study represents the first targeted effort that demonstrates the effectiveness of an eDNA metabarcoding approach for the detection and monitoring of elasmobranch communities.

## Results

### eDNA detection of elasmobranchs

**Figure 1:**
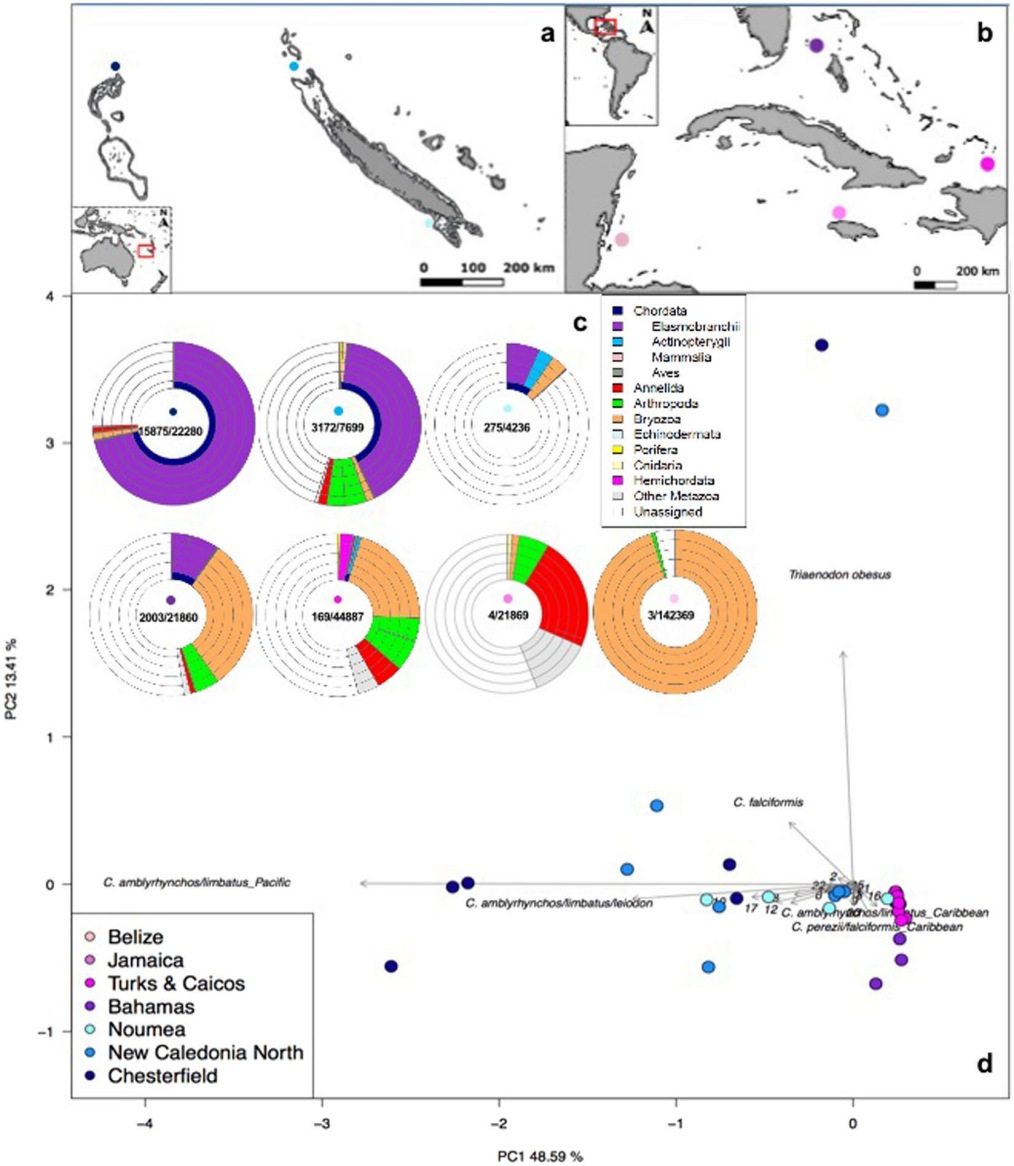
Map of New Caledonian (**a**) and Caribbean (**b**) sampling locations. The intensity of dot shading in all panels indicates the level of anthropogenic impact from ‘severely impacted’ (light pink/blue) to ‘least impacted’ (dark pink/blue). The krona-like plots (**c**) show the complete taxonomic assignment for each of the sampling locations (with elasmobranchs in purple). The different taxonomic levels are represented by the layers of rings, starting with phylum, for the innermost layer, and subsequently class, order, family and genus radiating outwards. In the centre of each location plot, the number of elasmobranch reads compared to the total number of filtered reads, is displayed. The Principal Component Analysis (PCA) (**d**) depicts the scattering of the samples containing elasmobranch reads, across the two biogeographic areas. The six most discriminating taxa are labelled in full, while the rest are indicated by numbers (following alphabetical order from lines 27–50 in Supplementary Table S2), namely: 1 = *C*. *acronotus*, 2 = *C*. *albimarginatus*, 4 = *C*. *amblyrhynchos/limbatus*_Caribbean, 6 = *C*. *brachyurus/perezii*, 8 = *C*. *melanopterus/cautus*, 9 = *C*. *leucas*, 10 = *C*. *obscurus/macloti/longimanus/galapagensis*, 12 = *C*. *perezii/falciformis_Pacific*, 13 = *C*. *plumbeus*, 14 = *C*. *plumbeus/altimus/sorrah*, 15 = *G*. *cuvier*, 17 = *N*. *brevirostris/acutidens*_Pacific, 18 = *R*. *porosus/terraenovae*, 20 = *S*. *mokarran*, 21 = *D*. *Americana*, 22 = *S*. *fasciatum*. Maps made with Natural Earth. Free vector and raster map data @ naturalearthdata.com.

A total number of 2,972,832 reads was obtained from an Illumina MiSeq run of pooled amplicon libraries, built from 55 Caribbean and 22 New Caledonian samples (Fig. 1a,b and Supplementary Table S1). A large part of the sequenced reads (80%) originated from non-specific amplification and were shorter than the target length. After sample assignment, quality and sequence-length filtering, 284,252 reads were left; of which 21,542 could be taxonomically assigned to elasmobranchs (Supplementary Table S1). The number of elasmobranch reads per sample ranged from 0 to 5,205 (Supplementary Table S1). After the removal of singletons (MOTUs in a sample that contained only one read), taxonomic assignment from the sampled locations (Fig. 1a,b) resulted in 22 elasmobranch molecular operational taxonomic units (MOTUs), of which 12 were detected in the Caribbean, 16 in New Caledonia and 9 in both locations. Krona-like plots (Fig. 1c) display the complete taxonomic assignment for each of the sampling locations, while a Principal Component Analysis (PCA) (Fig. 1d) depicts the scattering of all the samples containing elasmobranch reads across the two biogeographic areas. Even though several MOTUs are shared between the two regions, there is still a clear spread in MOTU (species) composition between New Caledonia and the Caribbean. No elasmobranch reads were detected in the two PCR negative controls that were performed and sequenced to detect potential contamination.

Using the 127-bp COI fragment, we did not find a wholly unequivocal correspondence between MOTUs and species; since some MOTUs had 100% sequence identity matches with more than one species in the BOLD database. Although this issue mostly pertained to the genus *Carcharhinus*, which is known to be taxonomically problematic and polyphyletic^52^, it also affected the less speciose *Rhizoprionodon* and *Negaprion*. Consequently, this has also resulted in MOTUs containing sequences from both Coral Sea and Caribbean species that share an identical 127 bp sequence. Thus, for a more reflective separation, these particular MOTUs have been split into their Pacific and Caribbean components (see Supplementary Table S2 for a complete data file of all reads per taxa, per sample).

### Elasmobranch diversity and read abundance patterns

**Figure 2:**
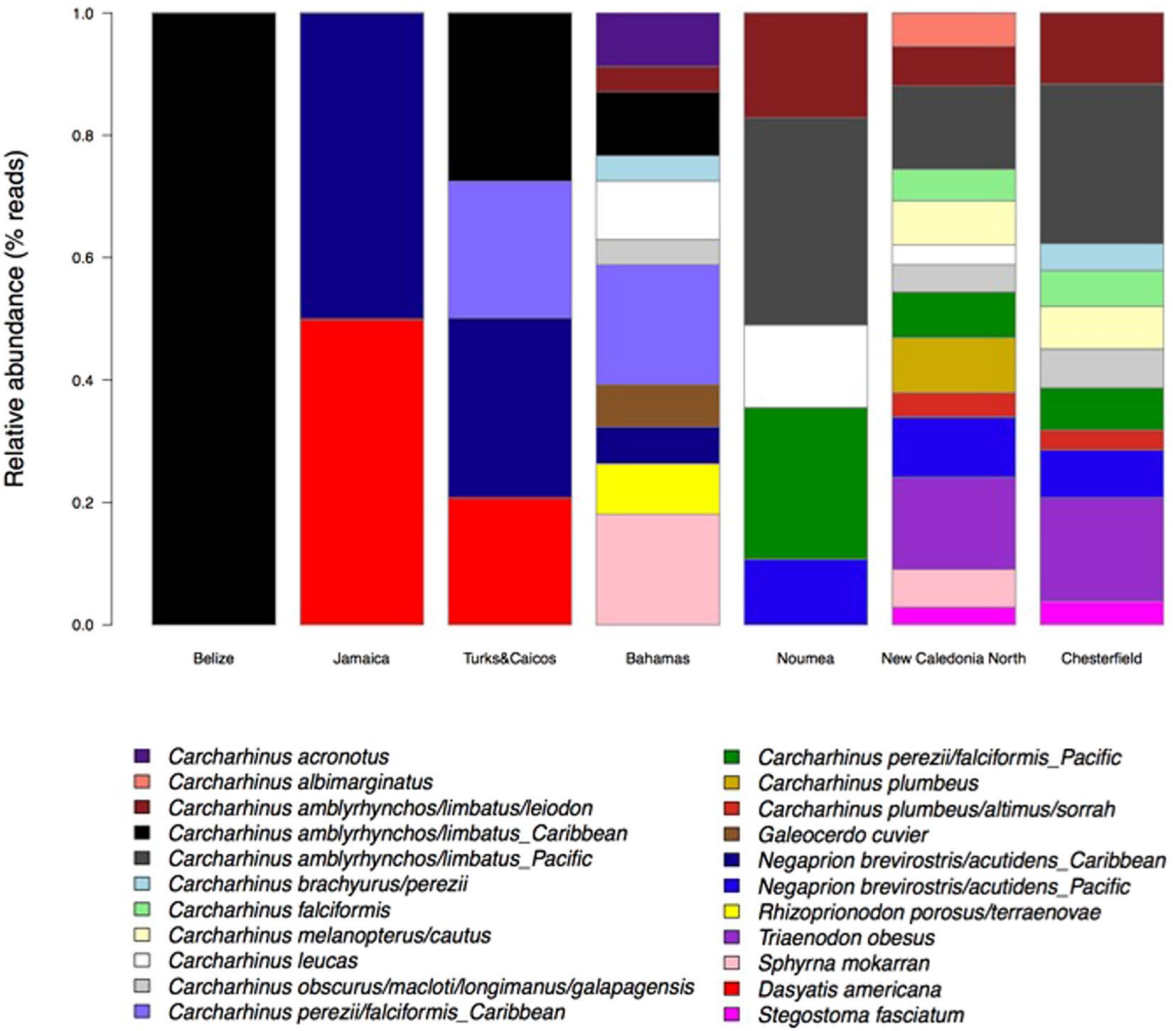
Bar plot showing the relative abundances of reads (fourth-root transformed) for every elasmobranch MOTU detected in the Caribbean and New Caledonian locations.

The Bahamas is the Caribbean sampling location least subjected to fishing pressure, as a result of its shark sanctuary status; hence, it displays the greatest elasmobranch diversity, composed of 11 different MOTUs (Fig. 2). In the samples from the locations most impacted by anthropogenic disturbances, Jamaica and Belize, only 2 and 1 elasmobranch MOTUs respectively, were detected. A similar pattern is apparent for the New Caledonian samples, where the highest diversity is found in the most remote and pristine locations, the Chesterfield Atolls (11 MOTUs) and New Caledonia North (14 MOTUs). Contrastingly, only 5 elasmobranch MOTUs were detected in the capital, Noumea, the most densely populated area of New Caledonia.

**Figure 3:**
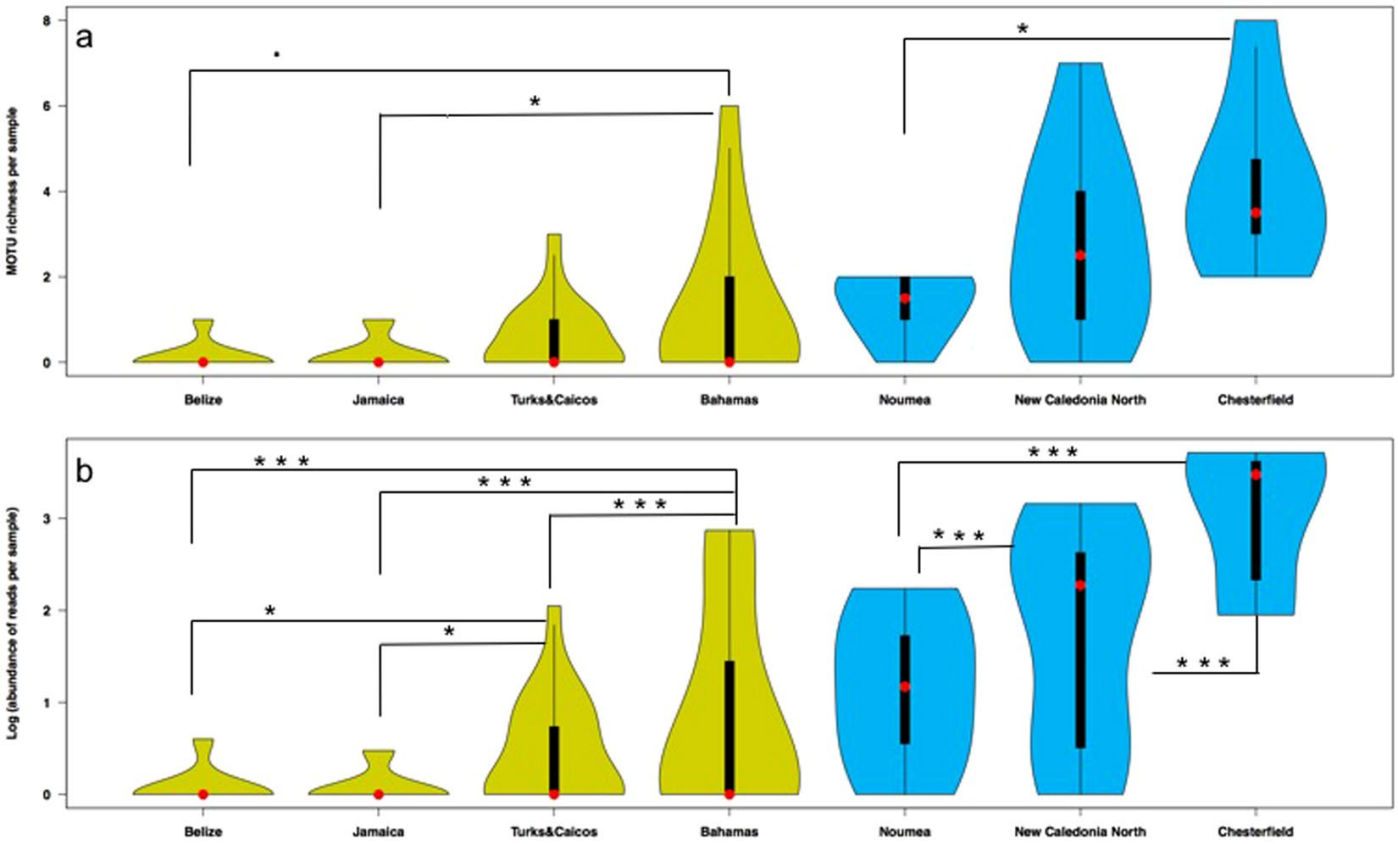
Violin plots showing (**a**) elasmobranch diversity (MOTU richness) and (**b**) abundance of reads per sample in the different locations from the Caribbean (green) and New Caledonia (blue). The shapes indicate the density distribution of the samples, extending from the minimum to the maximum observed values. The median values are indicated by the red dots. The thick black bars are the interquartile ranges. The thin black extending lines represent the 95% confidence intervals such that the values in the wider parts of the plots are more probable than those in the narrower parts. Per region, significant differences (P < 0.05) are indicated with asterisks. Asterisk significant codes: ***P < 0.001, **P < 0.01, *P < 0.05, ^•^P < 0.1.

The violin plots of MOTU richness (Fig. 3a) and read abundances (Fig. 3b) show how the different sample values are distributed, by comparing the variable sample size distribution across the different locations. The distribution of density (number of MOTUs, Fig. 3a), and abundance of reads, (Fig. 3b) is represented by the width of the plots. For both the Caribbean (green) and the New Caledonian (blue) locations, MOTU richness (Fig. 3a) increases from left to right, following the pattern of decreasing anthropogenic disturbance. While the three least impacted locations, the Bahamas, New Caledonia North and the Chesterfield atolls, show the greatest numbers of MOTUs in one sample (6, 7 and 8 MOTUs respectively), the violin plots for these locations show that richness is more equally spread across the different samples from Chesterfield (thus more samples containing multiple MOTUs); a sample is more likely to contain only 1 or 2 MOTUs in the Bahamas whereas in Chesterfield a sample is more likely to contain 4 or 5 MOTUs. Additionally, every sample from Chesterfield contains at least 3 elasmobranch MOTUs (for detailed MOTU richness per sample, see Supplementary Table S2). The abundance of reads (Fig. 3b) per location follows the same pattern: it increases in both Caribbean and New Caledonian locations, with a decreasing level of human impact. While both New Caledonia North and Chesterfield have samples that contain more than 1000 elasmobranch sequence reads, the number of samples with more than 1000 reads is greater in the more remote Chesterfield atolls. Additionally, Chesterfield is the only location without any samples with less than 80 sequence reads.

Generalized Linear Model (GLM) tests (using location as categorical factor) indicate significant differences in both diversity and read abundance between locations in both the Caribbean and New Caledonia. Pair-wise comparisons (post-hoc Tukey comparisons) show that for diversity (MOTU richness, Fig. 3a), significant differences are detected between the Bahamas and Jamaica (P = 0.039) and nearly significant differences between the Bahamas and Belize (P = 0.095). While diversity in New Caledonia is significantly different between Chesterfield and Noumea (P = 0.013). For read abundances (Fig. 3b), significant differences exist between the Bahamas and the other 3 Caribbean locations (P < 0.001). Additionally, read abundances in Turks & Caicos are significantly different from Jamaica (P = 0.033) and Belize (P = 0.047). In New Caledonia, all abundance comparisons are highly significant (P < 10^−10^).

**Figure 4:**
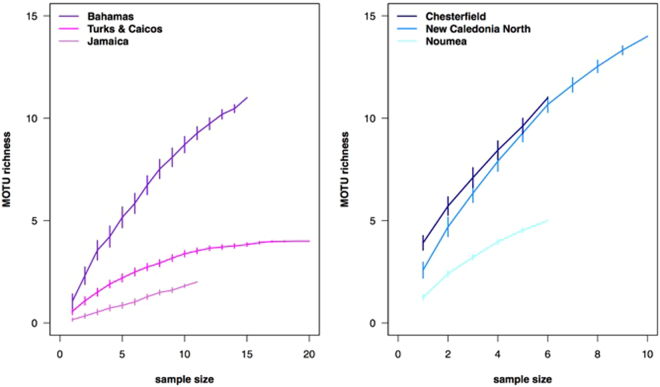
Species accumulation curves showing elasmobranch diversity (MOTU richness) as a function of the number of samples in the locations from the Caribbean (**a**) and New Caledonia (**b**). Error bars indicate standard errors after 100 permutations. Belize is absent from the plot as it contains only one elasmobranch MOTU.

Species accumulation curves are plotted for each location (Fig. 4). The curves show elasmobranch diversity (MOTU richness) as a function of the number of samples in the locations from the Caribbean (A) and New Caledonia (B). Error bars indicate standard errors after 100 permutations. The results show that none of the Caribbean (Fig. 4a) or New Caledonian (Fig. 4b) samples tend to reach a plateau in MOTU richness, although, with the exception of the Bahamas, the Caribbean slopes tend to flatten after N = 10. Non-saturation of species accumulation curves suggests that increased sampling effort would be desirable for capturing total diversity in each location, particularly in the naturally more diverse tropical Pacific.

## Discussion

Our results demonstrate that eDNA metabarcoding can be applied to assess elasmobranch species richness and, potentially, relative abundance in natural seawater samples, the two main components of ecological communities. The derived geographical patterns of diversity and abundance of shark eDNA sequence reads may be used for monitoring purposes and ultimately to inform conservation management and policy decisions. At the global/macro-scale, the detected elasmobranch MOTUs collectively separate the Caribbean and New Caledonian regions (Fig. 1d), dominated by *Carcharhinus perezii/Negaprion brevirostris* and *Carcharhinus amblyrhynchos / Triaenodon obesus*, respectively, with the exception of the most depauperate locations (e.g. Belize, Jamaica and Noumea) which are grouped in the center of the ordination plot. Additionally, the patterns of MOTU richness and abundance of sequence reads follow the level of anthropogenic impact in each location. Remote localities such as the Chesterfield atolls and protected areas such as Bimini, Bahamas show both the highest species richness and read abundance among our samples, whereas the less remote and non-protected locations show lower values for both diversity and abundance (Figs 1c, 2 and 3).

In marine ecosystems, the impact on living resources is often framed into the Malthusian theory of human density around such ecosystems^53^. Several studies have shown proximity to market to be the strongest predictor of overfishing on coral reefs^54–56^. A particular case study from New Caledonia has demonstrated that travel time from the market is a strong predictor of fish biomass, predator abundance and functional diversity on coral reefs^53,57^. Thus, remote locations such as the Chesterfield atolls, receive *de facto* protection due to their isolation^57^. The level of elasmobranch diversity and eDNA read abundance, sheds a new light on these wilderness areas that are already known to support high levels of fish biomass^51,57^.

Likewise, it has previously been shown that sharks on reefs in the Greater Caribbean mostly occur in areas with low human population density or in a few places where strong fishing regulations or conservation measures have been implemented^8^. While none of our Caribbean sampling locations is at more than 1 hour travel away from people, The Bahamas represents one of those locations whose elasmobranch populations in particular receive protection through effective conservation measures^48^.

Environmental DNA metabarcoding has a number of advantages compared to classical approaches for monitoring elasmobranchs. It is a minimally invasive and resource-effective technique. The eDNA sampling and metabarcoding protocols are easy to standardize and the molecular assignment does not require taxonomic expertise. Nevertheless, our findings also reveal a number of concerns that should be addressed in future developments of shark/elasmobranch eDNA metabarcoding approaches. First the taxonomic resolution of the final dataset is strongly dependent on the choice of markers; while the use of COI as a metabarcoding marker has previously been criticized^58^, owing to its high sequence variability, which may impair the design of truly universal primers and complicate bioinformatics analysis - it can also be argued that COI presents two major advantages over other potential markers. First, the steadily growing international effort, headed by the Consortium for the Barcode of Life (CBOL), to develop a public DNA barcoding database with curated taxonomy, greatly facilitates taxonomic assignment. The BOLD database (http://www.boldsystems.org/)^59,60^ currently includes >4 million sequences belonging to over 500,000 species, curated and identified by expert taxonomists. Secondly, the high mutation rate of COI enables identification at the species level, whereas the highly conserved sequences of other markers, such as 18S, make it often impossible to distinguish at the species or genus levels; and species-level identification is crucial for studies aimed at detecting rare species, such as is often the case for sharks.

Nevertheless, using the 127 bp elasmobranch specific COI fragment^46^, we still recorded some ambiguity in the taxonomic assignment of some species of the genera *Carcharhinus*, *Rhizoprionodon* and *Negaprion*, owing to the limited sequence variability within the amplicon. Consequently, the sequences of some MOTUs are 100% identical to individuals belonging to different species in the BOLD database. In our dataset, this is presented by several MOTUs belonging to either of two or more species. Furthermore, this has resulted in 3 MOTUs having 100% sequence identity for species occurring in New Caledonia, and species occurring in the Caribbean. One particular example is the *Carcharhinus amblyrhynchos/limbatus* MOTU. The bulk of sequences within this MOTU are from New Caledonia, 16730 compared to 146 in the Caribbean (Supplementary Table S2). In all probability, the sequence reads from New Caledonia belong to both *C*. *amblyrhynchos*, the grey reef shark (a species abundant in this area and also the species most often visually detected during sampling operations, Supplementary Table S1) and to *Carcharhinus limbatus*, the blacktip shark. Since *C*. *amblyrhynchos* does not occur in the Caribbean, the 146 reads of this MOTU in the Caribbean samples most likely belong to *C*. *limbatus*. Similar considerations apply to *Negaprion brevirostris/acutidens* and *Carcharhinus perezii/falciformis,* whose MOTUs are shared between New Caledonia (365 and 206 reads respectively) and the Caribbean (91 and 1045 reads respectively). *Negaprion brevirostris* (lemon shark) is native to the Americas whereas *N*. *acutidens* (sicklefin lemon shark) is widely distributed throughout the Indo-Pacific. *Carcharhinus falciformis*, the silky shark, is circumtropical, while the distribution of *Carcharhinus perezii* (Caribbean reef shark) is restricted to the tropical western Atlantic Ocean.

In order to resolve the issue with closely related species, it will be essential to design alternative primers that are able to amplify a longer fragment of the gene region, in addition to an improved reference database. In relation to this, it is clear that certain elasmobranch species present in the environment at the time of sampling could not be detected using the selected primer set, as their non-degenerate sequences contain mismatches with the binding regions of several species. This is epitomized by the case of the nurse shark, *Ginglymostoma cirratum*, which is an abundant species in the Caribbean and some individuals were visually observed at the time of sampling. Yet, eDNA sequence reads of *G*. *cirratum* were not detected in any of our Caribbean samples. Comparing the available *G*. *cirratum* COI sequences in public repositories with our primer sequences, it becomes apparent that two mismatches with the primer 3′ end are most likely responsible for the prevention of amplification of this species’ DNA from environmental samples. In silico mismatch statistics, comparing the 3′ half of the primers with full mitochondrial genome sequences available, are listed in Supplementary Table S4, for all elasmobranch Orders. This table shows that the primers are particularly suitable for amplifying Carcharhiniformes, which contains more than half of all shark species, including those of most ecological relevance for this study. However, all the elasmobranch orders may be amplified. Potential mismatch issues may be resolved by decreasing the specificity of the primer set by incorporating degenerate bases or inosine nucleotides. However, this approach may have the undesired effect of an increase in the number of reads belonging to non-target taxa getting amplified. Clearly, this trade-off between taxonomic resolution and non-target amplification needs to be well balanced prior to applying the eDNA metabarcoding approach for the purpose of informing conservation and management decisions. Here, we focused on large-scale differences and overall patterns in relation to anthropogenic influence, but in alternative contexts it may be necessary to attain greater taxonomic accuracy (e.g. endangered or invasive species); a challenge also faced by currently established ribosomal amplicon-based analysis of other vertebrates^45,61^.

Quantification of eDNA relating to species abundance could provide clues to habitat use and preference, thus identifying spatial conservation priorities such as home ranges and dispersal and migration corridors^62^. However, whether eDNA metabarcoding can provide quantitative estimates, particularly in the case of community-level abundance, remains a controversial issue. Although amplicon sequencing produces read counts that may contain valuable information about target species abundances^40,63^, the interpretation of the results of amplicon studies, in the context of quantitative ecology, is not straightforward and remains ambiguous^64^. This is in part because shedding rates between communities, species and individuals may differ. But also because the precise relationship between amplicon abundance and taxon abundance remains unknown and likely varies among taxa^63,65^, as it is argued that PCR products are not fully proportional to real abundances due to the fact that primer efficiency may vary among species templates (primer bias)^65,66^. While previous studies have shown positive rank correlations between species abundance and read abundance^38,43,63,67^, as of yet, no studies have been published, revealing evidence of a relationship between relative abundance of species within a community and their respective eDNA read abundances. And while currently no experiments have been performed to empirically verify the relationship between read abundance and community biomass for elasmobranchs in particular, our data show that read abundances are higher in the more pristine/remote sampling locations and that these patterns of read abundance are coherent with expectations that can be inferred from the contrasting levels of human impact/remoteness of the different locations, for both the Caribbean and New Caledonia (Fig. 3b). While for example the species diversities of New Caledonia North and Chesterfield are very similar (Fig. 2), eDNA read abundance is significantly higher in Chesterfield (Fig. 3b), suggesting that read abundance may be correlated with remoteness. As the relationships between eDNA and species abundance become clearer, the role of eDNA in estimating species abundance in both freshwater and marine environments is likely to become more valuable, increasing the potential of future eDNA applications in research and conservation.

Conservation and management of elasmobranch diversity relies on the effective monitoring of species across large oceanic areas. While direct observation and identification of individuals are often complicated, we have demonstrated that, despite the seemingly daunting task of probing vast stretches of ocean by collecting water samples, eDNA metabarcoding has great potential for developing into an objective and powerful elasmobranch assessment tool, applicable to a wide range of ecological goals, from the mapping of diversity gradients in response to environmental variation, to the monitoring of the effectiveness of spatial protection measures.

## Material and Methods

### Experimental Design

Aqueous eDNA samples were collected with interoceanic replication, to test for spatial marine protection as a predictor for elasmobranch diversity. During February and March of 2015 (Supplementary Table S1), samples were collected from four Caribbean locations impacted by various levels of anthropogenic pressures (Fig. 1b). Jamaica is known to have one of the most depauperate fish populations in the Caribbean and a severely extirpated elasmobranch fauna^68^; thus it was expected that Jamaica would sit at the lower end of the elasmobranch diversity range and read abundance. In Belize sampling took place around the partially submerged Glover’s Reef atoll, which is part of the Mesoamerican Barrier Reef. Even though this region has a relatively large number of marine reserves, including the ‘Glovers Reef Marine Reserve’, shark sightings in the Caribbean are quite rare and relatively few shark sightings occurred in the Mesoamerican Barrier Reef area during a previously conducted survey^8,69^.

In the Turks & Caicos Islands, where sampling took place around South Caicos, the establishment of a shark sanctuary is under consideration; however, the islands are currently still experiencing high fishing pressure^47^, which tends to disproportionally reduce densities of longer-lived, larger-bodied individuals^50^. At the other end, the nation of Bahamas is a designated shark sanctuary^48^ and as such, an area characterised by consolidated shark protection. The sampling was conducted around the islands of Bimini, which consequently boast an abundant and diverse elasmobranch fauna^70,71^.

In the tropical Pacific, eDNA samples were collected from three locations in New Caledonia (Fig. 1a), during September, October and November of 2015 (Supplementary Table S1). New Caledonia has a unique anthropogenic impact gradient from nearly pristine to significant levels of anthropogenic disturbance^57^. The most heavily impacted site is represented by the capital Noumea, the most densely populated area in New Caledonia^53^. However, most reefs near Noumea are no-take reserves and shark fishing is historically non-existent in New Caledonia so that near Noumea, shark populations may be healthier compared to many other impacted areas. Sampling sites north of the main island Grande Terre, ‘New Caledonia North’, represent the intermediate level of anthropogenic impact, being between 70–120 km removed from the nearest human settlement and a minimum of 500 km/15 hours travel time, from the Noumea fish market. The isolated Chesterfield atolls, 550 km northwest of Grande Terre (~35 h travel time from the Noumea fish market), are the most remote of all our sampling locations. These samples were expected to show the highest levels of elasmobranch diversity and eDNA read abundance. Within all 7 (Caribbean and New Caledonian) locations, samples were collected from between 6 and 20 different sites covering a variety of habitats (Supplementary Table S1 contains coordinates per site). A total of 55 samples from the Caribbean and 22 samples from New Caledonia were collected and analysed. Each sample consisted of 4 litres of sea water, collected by either a Kemmerer type water sampler or directly with a plastic collection bottle.

### Sample processing and DNA extraction

After collection, the water samples were individually covered and stored, in the dark and on ice, during transport to the local laboratory facilities. Vacuum filtration was carried out within two hours after collection. When it was not feasible to carry out filtration within two hours after collection, due to travel time to laboratory facilities, the samples were directly frozen after collection, until further processing. The sterile mixed cellulose esters (MCE) filters (Merck Millipore; 47 mm diameter; 0.45 µm pore size) containing sample filtrates were stored in 2.0 ml screw-cap microcentrifuge tubes containing silica beads. The silica beads function as a desiccator, drying out the filters and hence preventing the DNA from degrading. The sample filters were then stored at −20 °C until extraction. DNA was extracted from the filters with the Mo-Bio PowerSoil DNA Isolation Kit (www.mobio.com), following the manufacturers’ protocol. Purified extracts were assessed for DNA concentration in a Qubit fluorometer (Thermo Fisher Scientific).

### Contamination Control

Contamination of samples may occur anywhere from preparing sampling equipment and collecting the samples in the field (target DNA being carried unintentionally from one locality to another), to every subsequent step of sample preparation, extraction and analysis in the laboratory. Hence, strict adherence to contamination control was followed at all field and laboratory stages in order to prevent the occurrence of contamination, including the use of disposable gloves and single use-sterile collection bottles and filtration equipment, and the bleaching (50% bleach) of sampling devices and laboratory equipment and surfaces. Additionally, a dedicated controlled eDNA lab at the University of Salford, with separate rooms designated for the physical separation of eDNA extraction, pre-PCR preparations and post-PCR procedures, was used for all laboratory work. Moreover, to identify potential contamination, DNA extraction blanks (elution buffer from extraction kit) and PCR blanks were included.

### Library preparation and sequencing

For the amplification of eDNA metabarcoding markers, an elasmobranch specific COI primer set was used. This previously published primer set consisted of a novel reverse primer ‘Shark COI-MINIR’ 5′-AAGATTACAAAAGCGTGGGC-3′^46^ and two universal fish barcoding forward primers FishF2 5′-TCGACTAATCATAAAGATATCGGCAC-3′ and VF2 5′-TCAACCAACCACAAAGACATTGGCAC-3′^72^, yielding an amplicon of 127 bp^46^. For the multiplex Illumina sequencing run, we used 4 sets of 24 primers with attached 8-base sample-specific oligo-tags differing in at least 3 bases^73^. In order to increase variability of the amplicon sequences, a variable number (2, 3 or 4) of fully degenerate positions (Ns) was added at the beginning of each primer^74^. The full, sequenced PCR product, consisted then of 195 bp, including the amplicon, primers, sample tags and leading N’s.

For PCR amplification, a single step protocol was used, directly attaching the 8-base tagged primers. The PCR mix recipe was as follows: a total volume of 20 µl included 2 µl 10x buffer (BioLine), 0.6 µl 50 mM MgCl (BioLine), 0.5 µl of each of the 5 μM forward primers (Eurofins), 1 µl of the 5 µM reverse primer, 0.2 µl 10 mM dNTP mix (BioLine), 0.2 µl BioTaq DNA polymerase (5 u/μl, BioLine), a standardised amount (10 ng) of the filter-extracted eDNA template, and 13 µl sterile water. The PCR profile included an initial denaturing step of 95 °C for 15 min, 35 cycles of 94 °C 1 min, 52 °C 1 min and 72 °C 1 min and a final extension step of 72 °C for 5 minutes. The quality of all amplifications was assessed by electrophoresis, running the products through a 1.5% agarose gel stained with Gel Red (Cambridge Bioscience) and visualized on a UV light platform. All PCR products (including one replicate per sample and 2 PCR negative controls) were pooled into 4 multiplexed sample pools (each composed of 24 individually-tagged samples) and purified using MinElute columns (Qiagen). Four Illumina libraries were subsequently built from the four pools, using the NextFlex PCR-free library preparation kit (BIOO Scientific). The libraries were quantified using the NEBNext qPCR quantification kit (New England Biolabs) and pooled in equimolar concentrations along with 1% PhiX (v3, Illumina) serving as a positive sequencing quality control. The libraries with a final molarity of 8 pM were sequenced on an Illumina MiSeq platform in a single MiSeq flow cell using v2 chemistry (2 × 150 bp paired-ends).

### Bioinformatic and Statistical Analyses

The bioinformatic analysis was based on the OBITools metabarcoding software suite^75^. The pipeline used for data analysis is summarized in Supplementary Methods S3. Quality of the reads was assessed using FastQC. Paired-end reads were aligned using illuminapairedend and alignments with quality score >40 were kept. The aligned dataset was demultiplexed using ngsfilter. The length distribution of the demultiplexed reads showed a large percentage of short fragments (<95 bp), originating from non-specific amplifications and primer-dimer artefacts, which were not removed during the size selection step of library preparation. Thus, a length filter (obigrep) was applied to the aligned reads (120–135 bp) in order to select only the fragments with the correct target size. Reads containing ambiguous bases were also removed. The reads were subsequently dereplicated using obiuniq and a chimera removal step was performed using the uchime-denovo algorithm^76^ implemented in vsearch^77^. The MOTUs were delimited using the sumaclust algorithm^75^ with a constant similarity threshold of 99%. Taxonomic assignment of the representative sequences for each Molecular Operational Taxonomic Unit (MOTU) was performed using the ecotag algorithm^75^. We built a bespoke elasmobranch reference database using a custom R script for retrieving all COI elasmobranch sequences available from the BOLD database^60^, and subsequently selecting those that included our 127 bp target fragment. In order to add homologous sequences from other, non-elasmobranch taxa, an *in silico* PCR was performed against release R117 of the EMBL-EBI database using ecoPCR^78^. Subsequently, the obtained reference sequences were added to the elasmobranch sequences obtained from BOLD. These additional reference sequences were added to our elasmobranch database in order to avoid the incorrect assignment of amplified sequences, belonging to other taxa, to elasmobranchs. This combined reference database is available from http://github.com/metabarpark/Reference-databases. The final refining of the dataset included taxonomy clustering of MOTUs assigned to the same species.

Due to its high sensitivity, an additional challenge associated with eDNA metabarcoding, is the risk of contamination^79,80^, and hence the possibility of introducing false positive results. While it is certainly possible to detect a species present in a sample, represented by a single sequence read, it is not possible to completely exclude contamination (or sequencing error) as the potential cause of MOTUs containing only a single read, i.e. to dismiss single reads as potential false positives. Accordingly, we have opted for a more conservative approach and have removed all single read MOTUs from our samples.

All statistical analyses were performed in R v 3.3.0 (https://www.R-project.org/). The vegan package v. 2.4–0^81^ was used for the calculation of sample-based species accumulation curves. A generalized linear model approach was used for testing differences in MOTU richness and read abundances (square-root transformed) as a function of location, using the glm2 package v. 1.1.2^82^. The Poisson distribution family function was used for modelling the residuals and package multcomp^83^ was used for post-hoc Tukey comparisons. All custom R scripts are publicly available from http://github.com/metabarpark.

### Data and materials availability

All data needed to evaluate the conclusions in the paper are present in the paper and/or the Supplementary Materials. Original DNA sequences (after initial quality filtering, paired-end alignment and length filtering) can be found at: https://data.mendeley.com/datasets/489gckxbz3/1.

## Electronic supplementary material

Dataset 1

Dataset 2

Supplementary information

Dataset 3
