# Supplementary material for: Environmental DNA reveals tropical shark diversity in contrasting levels of anthropogenic impact
Source: Sci Rep. 2017 Dec 4;7:16886. doi: 10.1038/s41598-017-17150-2 (PMC5715122; doi:10.1038/s41598-017-17150-2)
Supplement: Supplementary file 3 — Supplementary information [file 41598_2017_17150_MOESM3_ESM.doc]

Supplementary Methods S3

**Environmental DNA reveals tropical shark diversity in contrasting levels of anthropogenic impact**

Judith Bakker^1^, Owen S. Wangensteen^1^, Demian D. Chapman^2^, Germain Boussarie^3,4^, Dayne Buddo^5^, Tristan L. Guttridge^6^, Heidi Hertler^7^, David Mouillot^3^, Laurent Vigliola^4^ & Stefano Mariani^1*^

^1^ Ecosystems & Environment Research Centre, School of Environment & Life Sciences, University of Salford, M5 4WT, UK.

^2^ Department of Biological Sciences, Florida International University, 11200 S.W. 8th Street, Miami, Florida

33199, USA.

^3^ MARBEC, UMR IRD-CNRS-UM-IFREMER 9190, Université Montpellier, Languedoc-Roussillon,

34095 Montpellier Cedex, France.

^4^ IRD (Institut de Recherche pour le Développement), Laboratoire d’Excellence Labex Corail, UMR IRD-UR-CNRS ENTROPIE, Centre IRD de Noumea, BP A5, 98800 Noumea Cedex, New Caledonia, France

^5^ University of the West Indies, Discovery Bay Marine Laboratory and Field Station, P.O. Box 35, Discovery Bay, St. Ann, Jamaica.

^6^ Bimini Biological Field Station Foundation, South Bimini Bahamas.

^7^The SFS Centre for Marine Resource Studies, Turks and Caicos Islands, UK.

Corresponding Author: Prof. Stefano Mariani, Tel: +44 (0)161-295-6913; Email: s.mariani@salford.ac.uk

**Metabarcoding pipeline for COI Elasmobranchii Fields et al. primers**

| **1. Paired-end alignment. Keep reads with quality > 40. Demultiplexing.**  illuminapairedend -r SHAK_S1_L001_R2_001.fastq SHAK_S1_L001_R1_001.fastq | obiannotate -S goodali:'"Good_SHAK" if score>40.00 else "Bad_SHAK"' | obisplit -t goodali  ngsfilter -t ngsfilter_SHAK_fields.tsv --fasta-output -u unidentified_SHAK.fasta Good_SHAK.fasta > SHAK.filtered.fasta |
| --- |
| **2. Filter sequences with lengths between 120 and 135 bp and with only 'ACGT'.**  obigrep -p 'seq_length>120' -p 'seq_length<135' -s '^[ACGT]+$' SHAK.filtered.fasta > SHAK.filtered_length.fasta |
| **3. Group unique seqs.**  obiuniq -m sample SHAK.filtered_length.fasta > SHAK.unique.fasta |
| **4. Change ids to a short index. Change format to vsearch. Remove chimeras.**  obiannotate --seq-rank SHAK.unique.fasta | obiannotate --set-identifier '"'SHAK'%09d" % seq_rank' > SHAK.new.fasta  owi_obifasta2vsearch -i SHAK.new.fasta -o SHAK.vsearch.fasta  vsearch --uchime_denovo SHAK.vsearch.fasta --sizeout --nonchimeras SHAK.nonchimeras.fasta --chimeras SHAK.chimeras.fasta --uchimeout SHAK.uchimeout.txt |
| **5. Cluster at 99% with sumaclust. Get cluster centers.**  sumaclust -t 0.99 -s count -p 10 SHAK.nonchimeras.fasta > SHAK.sumaclust99.fasta  obigrep -p 'cluster_center' SHAK.sumaclust99.fasta > SHAK.sumaclust99.centers.fasta |
| **6. Taxonomic assignment using ecotag.**  ecotag -d taxo_sharks -R d[b_Elasmobranchii_Bakker_et_al_2017.fasta](https://github.com/metabarpark/Reference-databases/blob/master/db_Elasmobranchii_Bakker_et_al_2017.fasta) SHAK.sumaclust99.centers.fasta > SHAK.ecotag.fasta |
| **7. Add taxa above order level.**  owi_add_taxonomy -i SHAK.ecotag.fasta -o SHAK.ecotag.fasta.annotated.csv |
| **8. Recount abundances by sample.**  obitab -o SHAK.sumaclust99.fasta > SHAK.sumaclust99.tab  owi_recount_sumaclust -i SHAK.sumaclust99.tab -o SHAK.sumaclust99.counts.csv |
| **9. Combine ecotag and abundance files.**  owi_combine -i SHAK.ecotag.fasta.annotated.csv -a SHAK.sumaclust99.counts.csv -o SHAK_all_MOTUs.csv |
| **10. Collapse MOTUs.**  owi_collapse -s 13 -e 88 -i SHAK_all_MOTUs.csv |
| **11. Curate the dataset manually.** |
| **12. Re-collapse MOTUs after curating.**  owi_collapse -s 13 -e 88 -i SHAK_all_MOTUs_curated.csv |
