# Supplementary material for: Environmental DNA reveals tropical shark diversity in contrasting levels of anthropogenic impact
Source: Sci Rep. 2017 Dec 4;7:16886. doi: 10.1038/s41598-017-17150-2 (PMC5715122; doi:10.1038/s41598-017-17150-2)
Supplement: Supplementary file 4 — Dataset 3 [file 41598_2017_17150_MOESM4_ESM.doc]

Supplementary table S4

**Environmental DNA reveals tropical shark diversity in contrasting levels of anthropogenic impact**

Judith Bakker^1^, Owen S. Wangensteen^1^, Demian D. Chapman^2^, Germain Boussarie^3,4^, Dayne Buddo^5^, Tristan L. Guttridge^6^, Heidi Hertler^7^, David Mouillot^3^, Laurent Vigliola^4^ & Stefano Mariani^1*^

^1^ Ecosystems & Environment Research Centre, School of Environment & Life Sciences, University of Salford, M5 4WT, UK.

^2^ Department of Biological Sciences, Florida International University, 11200 S.W. 8th Street, Miami, Florida

33199, USA.

^3^ MARBEC, UMR IRD-CNRS-UM-IFREMER 9190, Université Montpellier, Languedoc-Roussillon,

34095 Montpellier Cedex, France.

^4^ IRD (Institut de Recherche pour le Développement), Laboratoire d’Excellence Labex Corail, UMR IRD-UR-CNRS ENTROPIE, Centre IRD de Noumea, BP A5, 98800 Noumea Cedex, New Caledonia, France

^5^ University of the West Indies, Discovery Bay Marine Laboratory and Field Station, P.O. Box 35, Discovery Bay, St. Ann, Jamaica.

^6^ Bimini Biological Field Station Foundation, South Bimini Bahamas.

^7^The SFS Centre for Marine Resource Studies, Turks and Caicos Islands, UK.

Corresponding Author: Prof. Stefano Mariani, Tel: +44 (0)161-295-6913; Email: s.mariani@salford.ac.uk

Results from the in silico PCR study performed with the primers used for this study, on all the elasmobranch orders in our reference database. The in silico mismatch statistics, compare the 3’ half of the primers with the full elasmobranch mitochondrial genome sequences available. N: the number of species with mitochondrial genome, per order.
